# 3D reconstruction method based on second-order semiglobal stereo matching and fast point positioning Delaunay triangulation

**DOI:** 10.1371/journal.pone.0260466

**Published:** 2022-01-25

**Authors:** Yongbing Xu, Kaixiang Liu, Jinyan Ni, Qingwu Li

**Affiliations:** 1 Shandong Survey and Design Institute of Water Conservancy, Jinan, China; 2 College of Internet of Things, Hohai University, Changzhou, China; Universidade Federal de Uberlandia, BRAZIL

## Abstract

Binocular vision uses the parallax principle of the human eye to obtain 3D information of an object, which is widely used as an important means of acquiring 3D information for 3D reconstruction tasks. To improve the accuracy and efficiency of 3D reconstruction, we propose a 3D reconstruction method that combines second-order semiglobal matching, guided filtering and Delaunay triangulation. First, the existing second-order semiglobal matching method is improved, and the smoothness constraint of multiple angle directions is added to the matching cost to generate a more robust disparity map. Second, the 3D coordinates of all points are calculated by combining camera parameters and disparity maps to obtain the 3D point cloud, which is smoothed by guided filtering to remove noise points and retain details. Finally, a method to quickly locate the insertion point and accelerate Delaunay triangulation is proposed. The surface of the point cloud is reconstructed by Delaunay triangulation based on fast point positioning to improve the visibility of the 3D model. The proposed approach was evaluated using the Middlebury and KITTI datasets. The experimental results show that the proposed second-order semiglobal matching method has higher accuracy than other stereo matching methods and that the proposed Delaunay triangulation method based on fast point location requires less time than the original Delaunay triangulation.

## 1 Introduction

Binocular simulation of human vision matches the corresponding pixels of image pairs to obtain disparity maps and calculates the 3D coordinate of each point in the 3D scene. This process has wide applications in 3D reconstruction, industrial inspection, robotics navigation and virtual reality. However, binocular vision still has great limitations in obtaining the 3D information of a scene, although its ability to roughly calculate the depth range is sufficient for humans. For 3D reconstruction tasks, the approximate depth range is not sufficient. Recovering a realistic 3D scene in a computer requires accurate 3D coordinates. In addition, efficient and realistic texture mapping is also indispensable. We improved the key components of the 3D reconstruction pipeline based on binocular vision, including stereo matching, point cloud filtering and triangulation. Finally, the accuracy and visibility of 3D reconstruction are improved. Specifically, the proposed method includes a semiglobal stereo matching method based on second-order smoothness constraints, a point cloud smoothing method based on guided filtering, and a Delaunay triangulation method based on fast point positioning.

Stereo matching is vital in binocular vision, and directly influences the 3D reconstruction results. Stereo matching methods can be divided into four steps: cost calculation, cost aggregation, disparity calculation and disparity refinement [1]. The methods are classified into three categories according to cost aggregation: local matching methods [2–5], global matching methods [6–11], and semiglobal matching(SGM) methods [12]. The proposed second-order semiglobal stereo matching method aggregates multiple cost loss functions to enhance the robustness of the method, and the matching error of the method is decreased by pooling cost loss in different directions.

The 3D coordinates of all points are calculated according to camera parameters and disparity maps. The 3D point cloud obtained by the above steps contains considerable noise, and needs to be smoothed. Point cloud filtering methods include bilateral filtering [13] and guided filtering [14]. Bilateral filtering combines spatial proximity and intensity similarity, which can not only remove the noise points but also preserve the detailed features. However, the method has higher computational complexity. Guided filtering is a local linear filtering method with lower computational complexity than bilateral filtering that introduces guided images. The method can achieve good results. To restore the visual surface of a 3D scene, surface reconstruction and texture mapping are also required. Surface reconstruction methods include the distance field contour surface method [15], Poisson reconstruction [16], and Delaunay triangulation [17]. The distance field contour surface method derives the initial tangent plane from the K-nearest neighborhood points and extracts the contour surface by forming the distance field according to normal vector uniformity. Poisson reconstruction adopts invisible fitting, and obtains an invisible equation corresponding to the point cloud model by solving the Poisson equation. Delaunay triangulation connects 3D points into triangles according to certain rules and has high stability. After reconstructing the surface of the scene, texture mapping is performed to enhance the realism of the 3D model.

In this paper, we propose a 3D reconstruction method based on binocular stereo matching and Delaunay triangulation based on fast point positioning to improve the accuracy and efficiency of 3D reconstruction. The contributions of this paper are as follows: (1) A semiglobal stereo matching method combining multiple matching costs is proposed to obtain disparity maps, which are applied to subsequent 3D reconstruction. (2) A 3D point cloud smoothing method based on guided filtering is used to effectively remove noise points and retain the details of the 3D point cloud. (3) The Delaunay triangulation method based on fast point positioning is proposed to accelerate the Delaunay triangulation. Finally, the surface of the point cloud is accurately reconstructed, and the 3D reconstruction of the binocular image pair is realized.

The remainder of this paper is organized as follows: Section 2 introduces related work similar to the proposed method in the field of stereo matching and Delaunay triangulation. Section 3 introduces the proposed 3D reconstruction method. Section 4 discusses the overall evaluations and discussions on the proposed method. Section 5 draws conclusions and outlines directions for future research.

## 2 Related work

The 3D reconstruction method based on binocular stereo matching obtains the parallax of the left and right images through stereo matching to restore depth information, and then constructs a surface triangle mesh and implements texture mapping to achieve 3D reconstruction. In the following, the latest developments in related research will be explained in terms of stereo matching and the construction of surface triangular meshes.

### 2.1 Stereo matching

Early stereo matching was based on feature point matching [18–20]. The sparse disparity map that was obtained must be converted into a dense disparity map through interpolation calculation, however, the interpolation process is complicated. Feature extraction and positioning have a great influence on the matching result. The current sparse stereo matching is mostly applied to tasks such as camera pose estimation in SLAM. To avoid the complex process and errors caused by interpolation, most of the existing methods for recovering the 3D information of a scene directly obtain the dense disparity map [21, 22].

The current research on stereo matching mainly focuses on matching strategies, matching cost calculation and cost aggregation. Stereo matching methods are divided into global methods [7, 9], local methods [4, 5] and semiglobal methods [12] according to the different matching cost aggregation processes. The global method is more robust to occlusion and weak texture areas, which is more complex and time-consuming. Compared with the global method, the local method is faster but less accurate. The semiglobal method(SGM) converges matching costs from multiple directions to achieve a balance of accuracy and efficiency and is widely used in vision-based 3D reconstruction. SGM uses single-pixel mutual information(HMI) as the matching cost and performs one-dimensional energy minimization along multiple directions to approximately replace the two-dimensional global energy minimization. Woodford et al. [23] proposed a quadratic pseudo-Boolean optimization to calculate the second-order smoothness constraint and achieved superior performance in experiments. The proposed second-order semiglobal stereo matching method combining multiple matching costs can take into account the time efficiency and robustness of weak texture regions.

### 2.2 Mesh reconstruction based on triangulation

Triangulation divides discrete and disordered point clouds into mesh grids in 3D space. Usually, the point cloud is projected onto a two-dimensional plane to form a discrete point set in the plane area. Then, an irregular triangulated network of the point set is constructed. Among the methods for generating triangle meshes, the Delaunay triangulation method is the best, as it avoids the appearance of ill-conditioned triangles. Common methods for constructing Delaunay triangular meshes include the divide and conquer method [24], point-by-point insertion method [25], sweep surface line method [26], and triangulation growth method [27]. The triangulation growth method has been gradually eliminated due to its low efficiency. The divide-and-conquer method recursively divides the set of points and merges it level by level from bottom to top to generate the final triangulation. This is the most efficient method, but due to its use of recursion, the running process consumes considerable memory and cannot process a large amount of data. The point-by-point interpolation method constructs a convex polygon containing all points, generates the initial triangulation, and inserts the remaining points one by one. This method occupies a small amount of memory and can handle a large amount of data. However, as the triangle increases, the operating efficiency of the insertion point gradually decreases. The proposed method of quickly locating insertion points can improve the efficiency of Delaunay triangulation based on point-by-point insertion.

## 3 The proposed method

### 3.1 Matching cost of semiglobal stereo matching

In the actual scene, the binocular image has large differences in the gray value of pixels near the matching point due to inconsistent light intensity, camera exposure, or radiation intensity on the surface of the object. In the proposed method, the census metric and the gradient metric are used as the matching cost. The census transformation reflects the local structural features of the matching point domain, and we denote the census transformation as *T*(*p*), where *p* represents the currently matched pixel. In Formula 1, *C*_*census*_(*p*, *d*) represents the census metric between the pair of matching points with the disparity value *d* in the binocular image pair. This is obtained by calculating the Hamming distance between *T*(*p*) and *T*(*p*_*d*_).

$$\begin{array}{c} C_{census}\left(p,d\right)=Hamming\left[T\left(p\right),T\left(p_{d}\right)\right] \end{array}$$ (1)

The gradient metric *C*_*gradient*_(*p*, *d*) can be obtained from Formula 2, where *C*_*gradient*_(*p*, *d*) represents the gradient metric of a matching point pair with a disparity *d*. ∇*I*(*p*) is the gradient value at pixel point *p*, which is obtained by the Sobel operator.

$$\begin{array}{c} C_{gradient}\left(p,d\right)=\nabla I_{l}\left(p\right)-\nabla I_{r}\left(p_{d}\right) \end{array}$$ (2)

**Fig 1:**
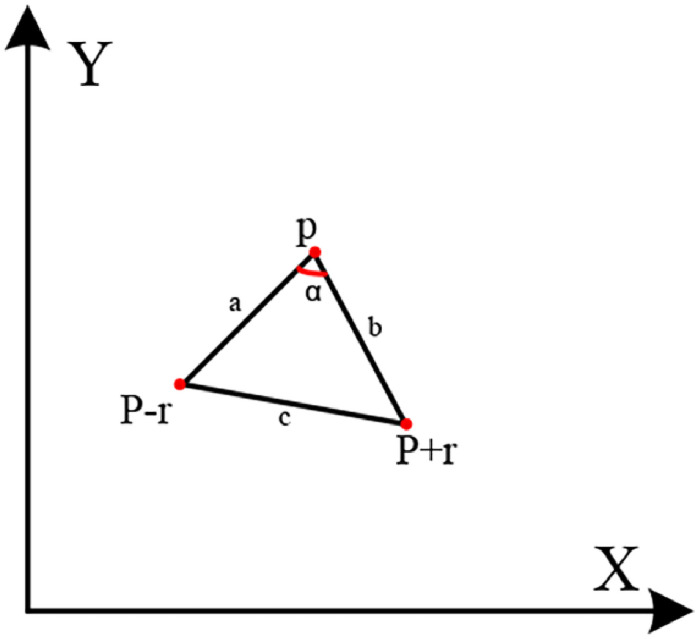
Angular direction of pixel *p*.

Since both the census metric and the gradient metric are cost metrics for a single pixel, they will be mismatched due to the effects of noise and lighting. The proposed second-order smoothness-constrained stereo matching cost aggregation method based on multidirectional angles can improve the matching accuracy of weak texture regions. This is achieved by constraining the parallax difference in multiple angle directions to adapt to inclined or curved surfaces. In Fig 1, the direction *r* of pixel *p* is represented as a triangular area composed of pixels *p*, *p* − *r*, and *p* + *r*. Pixel *p* in direction *r* is expressed as:

$$\begin{array}{c} \alpha_{r}\left(p\right)=arc\text{cos}(\frac{a^{2}+b^{2}+c^{2}}{2ab}) \end{array}$$ (3)

Formula 4 gives the definition of the disparity smoothness constraint *P*_3*r*_(⋅) of pixel *p* in direction *r*. When the angle *α* of direction *r* is small, the smoothness constraint is increased to maintain the discontinuity of the parallax; when the angle *α* of direction *r* is large, the smoothness constraint is reduced to adapt to the inclined or curved plane;

$$\begin{array}{c} P_{3r}\left(p\right)=\left[\frac{\pi }{\alpha_{r}\left(p\right)}-1.0\right]\cdot \tau \end{array}$$ (4)

In Formula 4, *τ* is the threshold used to prevent the parallax smoothness constraint from falling into the local minimum. The final second-order smoothness constraint is defined as:

$$\begin{array}{c} V\left(d,d^{\prime }\right)=\{\begin{array}{cc} P_{3r}\left(p\right) & if\;|d-d^{\prime }|=0\\ P_{1}+P_{3r}\left(p\right) & if\;|d-d^{\prime }|=1\\ P_{2}+P_{3r}\left(p\right) & if\;|d-d^{\prime }|>1 \end{array} \end{array}$$ (5)

where *d* is the disparity of pixel *p* and *d*′ is the disparity of pixel *p* − *r*. *P*_1_ and *P*_2_ are two constants, *P*_2_ > *P*_1_. The *P*_2_ penalty is applied to pixel *p* with large parallax smoothness to adapt to the inclined or curved surface; the *P*_1_ penalty applied to pixel *p* with small parallax smoothness to preserve the discontinuity of the edge. Therefore, the final matching cost must also make the cost aggregation of the current pixels affected by all pixels in multiple directions. Eight directions are selected from around the current pixel, and the matching cost in each direction is calculated using dynamic programming methods. Finally, the parallax is determined by the WTA(Winner takes all) rule. The path cost of a single pixel in a certain direction is defined as follows:

$$\begin{array}{c} \begin{array}{cc} L_{r}\left(p,d\right) & =C\left(p,d\right)+min[L_{r}\left(p-r,d\right),V\left(d,d\right),L_{r}\left(p-r,d-1\right)+V\left(d,d-1\right),\\ & L_{r}\left(p-r,d+1\right)+V\left(d,d+1\right),\underset{i}{min}L_{r}\left(p-r,i\right)+V\left(d,dmp\right)]-\underset{k}{min}L_{r}\left(p-r,k\right) \end{array} \end{array}$$ (6)

where $dmp=arg\underset{i}{min}L_{r}\left(p-r,i\right)$. In Formula 6, *L*_*r*_(*p*, *d*) represents the path cost of a single pixel in direction *r*, *d* is the parallax of the pixel, and *C*(*p*, *d*) represents the aggregation of *C*_*census*_(*p*, *d*) and *C*_*gradient*_(*p*, *d*).

### 3.2 3D reconstruction based on guided filtering and Delaunay triangulation

**Fig 2:**
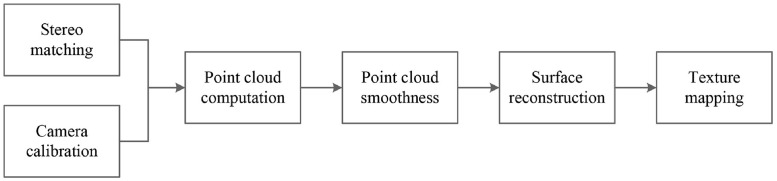
Flow chart of the 3D reconstruction method.

The flowchart of the proposed 3D reconstruction method is shown in Fig 2. It includes point cloud computation, point cloud smoothing, surface reconstruction, and texture mapping. The point cloud is obtained by combining the disparity maps and camera parameters, the disparity maps are obtained by stereo matching and the camera parameters are obtained by camera calibration. The point cloud is smoothed by filtering to remove the noise points. Finally, surface reconstruction and texture mapping are performed to acquire an authentic 3D model. This section focuses on point cloud smoothing and surface reconstruction during 3D reconstruction.

#### 3.2.1 Point cloud smoothing based on guided filtering

Due to errors in camera calibration and stereo matching, the obtained 3D point cloud includes much noise. Therefore, the point cloud must be smoothed before surface reconstruction. This section adopts guided filtering to smooth the point cloud. Guided filtering is a local linear filter method that effectively removes noise points and preserves the detail features. Guided filtering introduces the guiding image *G*. Here, *G* is the input itself and the relationship between the output image *O* and the input image *I* can be obtained as follows:

$$\begin{array}{c} O\left(p\right)=\sum_{q\in W_{p}}W_{pq}\left(G\right)I\left(q\right) \end{array}$$ (7)

where, *q* is the neighborhood pixel of pixel *p*, and the weight *W*_*pq*_(*G*) is defined as follows:

$$\begin{array}{c} W_{pq}\left(G\right)=\frac{1}{{\left|w\right|}^{2}}\sum_{\left(p,q\right)\in W_{k}}\left\{1+\frac{\left[G\left(p\right)-\mu_{k}\right)\left(G\left(q\right)-\mu_{k}\right]}{\sigma_{k}^{2}+\varepsilon }\right\} \end{array}$$ (8)

where, *μ*_*k*_ and $\sigma_{k}^{2}$ are the mean and variance of all pixels’ gray value in the window *w*_*k*_, |*w*| is the size of the window,and *σ* is an adjusting parameter. Guided filtering is introduced to 3D point clouds, and the original point cloud and smoothed point cloud satisfy the local linear relationship in depth. After guided filtering, all the vertices move along their normal direction, which is defined as follows:

$$\begin{array}{c} V^{\prime }\leftarrow V+d\cdot n \end{array}$$ (9)

where, *V* is a vertex in the point cloud, *V*′ is the vertex after smoothing by guided filtering, *d* is a weighting factor. The normal direction *n* of vertex *V* is calculated by local surface fitting. A plane is established to estimate the local geometry of the vertex *V*, and is defined as follows:

$$\begin{array}{c} F(x,y,z)=Ax+By+Cz+D=0 \end{array}$$ (10)

where, *A*, *B*, *C* and *D* are plane parameters. The KD-tree algorithm is adopted to select ten points near vertex *V* as the neighborhood spatial points. The coordinates of these points are substituted into Formula 10 so that the plane parameters can be estimated by the least squares method. Then, the updated normal direction is obtained as follows:

$$\begin{array}{c} n=(\frac{\partial F}{\partial x},\frac{\partial F}{\partial y},\frac{\partial F}{\partial z}) \end{array}$$ (11)

The weighting factor *d* is determined by the depth similarity between the center point and the local neighborhood points. The depth similarity refers to the distance vector that is projected onto the normal direction of the center point and is defined as follows:

$$\begin{array}{c} d=\frac{1}{{\left|s\right|}^{2}}\sum_{\left(p,q\right)\in S_{V}}\left[1+\frac{\left(<n,V-p>-\mu_{v}\right)\left(<n,V-q>-\mu_{v}\right)}{\sigma_{V}^{2}+\varepsilon }\right] \end{array}$$ (12)

where *S*_*V*_ is a neighborhood point set centered on vertex *V*, *p* and *q* are the neighborhood points of vertex *V*, and *S* is the size of set *S*_*V*_. <*n*, *V* − *p*> and <*n*, *V* − *q*> indicate the depth similarity between the two points *p* and *q* and vertex *V*. *μ*_*v*_ and *σ*_*v*_ indicate the mean and variance of all points’ depth similarity in the set *S*_*V*_, respectively.

#### 3.2.2 Projection-based Delaunay triangulation

**Fig 3:**
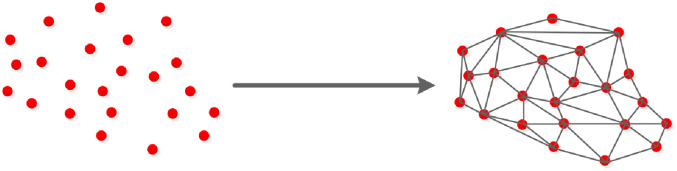
Triangulation.

To improve the visibility of the 3D model, a surface patch must be added on the point cloud. The surface patch can be any shape, generally a triangle or quadrangle is selected. Since a triangle is the smallest unit that constitutes the plane, it does not easily deform during the rotation process. Therefore, a triangle is selected as the basic unit to perform triangulation. As shown in Fig 3, projection-based Delaunay triangulation can be roughly divided into the following three steps:

1. Map a 3D point cloud to a 2D plane using orthogonal projections based on the normal direction.
2. Triangulate the 2D point set obtained from the mapping according to Delaunay criterion to determine the topological connection relationship between points.
3. Determine the topological connection between the original 3D points according to the topological connection relationship of the projection points in the plane. The obtained triangular mesh is the reconstructed surface model.

**Fig 4:**
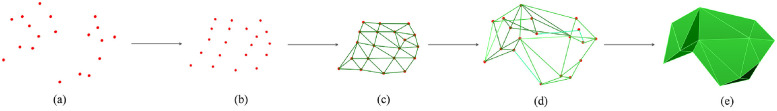
Flow chart of the projection-based Delaunay triangulation. (a) 3D point set (b) plane point set (c) plane triangulation (d) 3D triangulation (e) texture mapping.

Among the triangulation methods, Delaunay triangulation has the best mathematical features such as the closest, uniqueness, optimality, most regular, and convex polygon surface, so it is adopted for surface reconstruction. A projection-based Delaunay triangulation method is performed. First, the 3D point set is mapped to the *xOy* plane. Second, the plane point set is triangulated according to the Delaunay triangulation method based on the Bower-Watson algorithm [28]. Finally, the plane triangle mesh is mapped to the 3D space to generate a 3D model. Then, texture mapping is performed to restore the surface features of the 3D model. A flow chart of the projection-based Delaunay triangulation method is shown in Fig 4.

**Fig 5:**
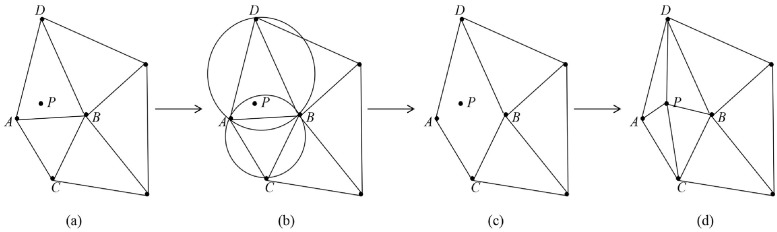
Flow chart of the Bowyer-Watson algorithm. (a) inserting the point *P* (b) searching for the influence triangles (c) deleting common edge *AB* (d) forming triangles.

The Bowyer-Watson method is currently the most common point-by-point insertion method, as shown in Fig 5. First, a supertriangle containing all of the scatter points is constructed. Second, each point is inserted in turn, and the influence triangles containing the point are found. Third, the common edges of the influence triangles are deleted, and the point and all the vertices of the influence triangles are joined. Finally, the unique triangular mesh model is obtained by adjusting the diagonal lines until all the points are inserted.

**Fig 6:**
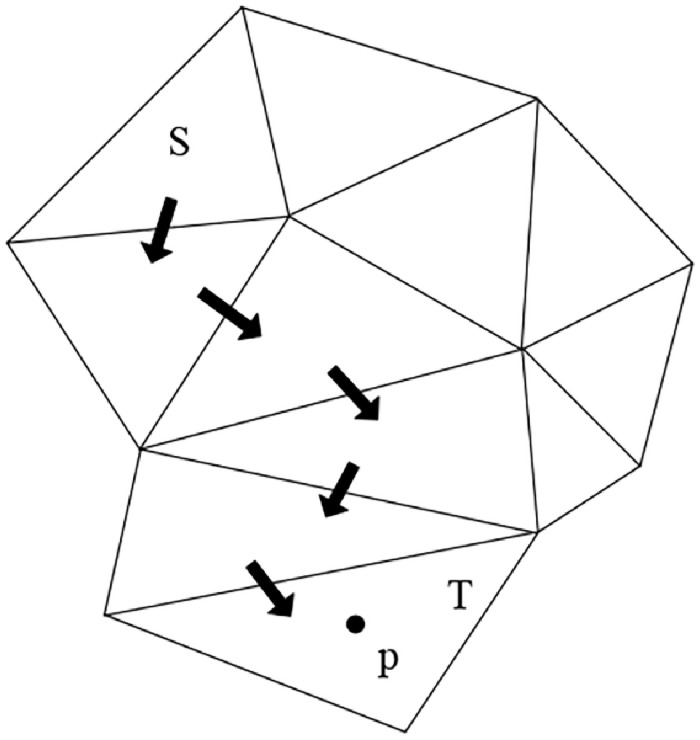
Method of quickly locating insertion point based on direction search.

In the process described above, the triangle containing the insertion point and the influence range of the insertion point must be found every time a point is inserted. The original Bowyer-Watson method needs to traverse the edges and vertices of all triangles. As the triangle increases, the time cost of these two steps will increase significantly. A method for quickly locating the insertion point based on direction search is proposed, which can quickly locate the triangle containing the point to be inserted. In the following, *G* indicates the barycentric of the triangle and *P* indicates the point to be inserted. Take the newly generated triangle as the initial triangle. Starting from the initial triangle, the search direction is determined by the relative position of *G* and *P*. When *G* and *P* are on different sides of a certain edge, the next triangle to be searched is the triangle adjacent to this edge. Stop searching when *G* and *P* are on the same side of the three edges of the triangle. This triangle contains the point to be inserted. Fig 6 shows the direction search method.

In Fig 6, *P* is the point to be inserted, *S* is the initial triangle, and *T* is the target triangle. In terms of implementation, the vertices of the triangle are arranged clockwise, such that the barycentric of the triangle is always on the right side of the edge. It is only necessary to determine whether the point to be inserted is on the left edge. For some special cases:

1. The point to be inserted is on a certain edge of the triangle: it is regarded as inside the triangle.
2. The point to be inserted is the vertex of the triangle: do not insert the point.
3. The point to be inserted is on the extension line of a certain edge of the triangle: it is considered to be on the same side of the edge as the barycentric. Continue to evaluate the next edge.

The method of quickly locating the insertion point based on direction search can be used to locate the triangle containing the points very quickly due to the point-by-point insertions according to the principle of spatial proximity.

## 4 Results and discussion

To verify the effectiveness of the proposed method, the C++ language is combined with OpenCV and OpenGL to realize the proposed method. The hardware platform is an Intel Core i5–4210H@2.90GHz. The Middlebury dataset [29, 30] and KITTI dataset [31] are adopted to evaluate the performance of the proposed method, and the depth information is compared with the ground truth to perform quantitative analysis of the reconstruction performance. The scene of the Middlebury dataset is an indoor environment, so a qualitative analysis of 3D reconstruction visualization for the Middlebury dataset is selected. In addition, our results are evaluated on the KITTI 2012 and KITTI 2015 benchmarks to obtain a quantitative analysis of the reconstruction effect. The contrast methods have certain similarities with the proposed semiglobal stereo matching method. Second, we compare the proposed Delaunay triangulation of fast point positioning with the original Delaunay triangulation-based on insertion points to show the speed advantage of the proposed fast point location method.

### 4.1 Middlebury dataset

**Fig 7:**
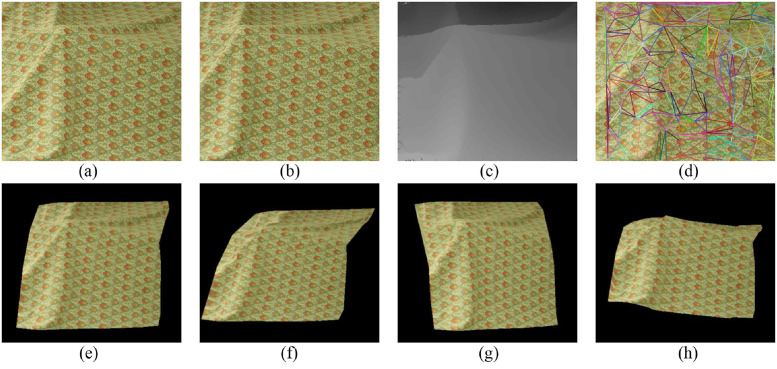
3D reconstruction experiment of scene Cloth1. (a) left image, (b) right image, (c) disparity image, (d) Delaunay triangulation, (e) view 1, (f) view 2, (g) view 3, (h) view 4. (a) and (b) are republished from [vision.middlebury.edu/stereo/] under a CC BY license, with permission from [D. Scharstein], original copyright [2002].

**Fig 8:**
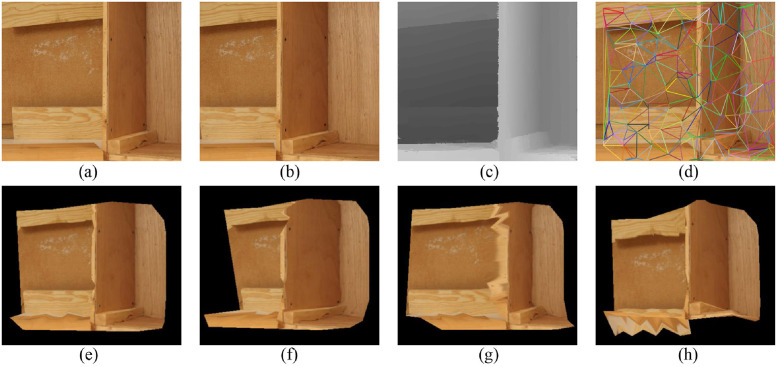
3D reconstruction experiment of scene Wood1. (a) left image, (b) right image, (c) disparity image, (d) Delaunay triangulation, (e) view 1, (f) view 2, (g) view 3, (h) view 4. (a) and (b) are republished from [vision.middlebury.edu/stereo/] under a CC BY license, with permission from [D. Scharstein], original copyright [2002].

**Fig 9:**
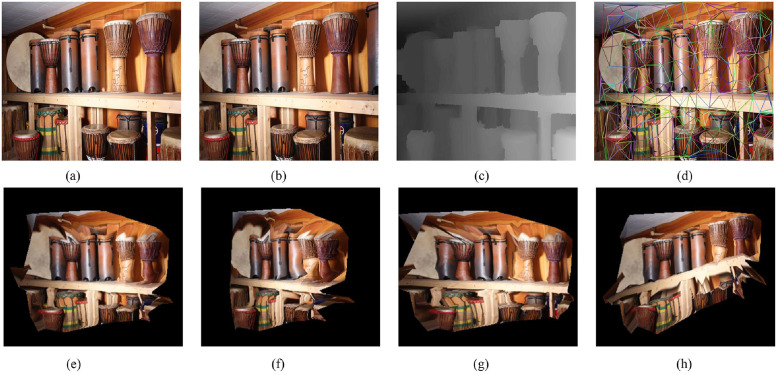
3D reconstruction experiment of scene Djembe. (a) left image, (b) right image, (c) disparity image, (d) Delaunay triangulation, (e) view 1, (f) view 2, (g) view 3, (h) view 4. (a) and (b) are republished from [vision.middlebury.edu/stereo/] under a CC BY license, with permission from [D. Scharstein], original copyright [2002].

**Fig 10:**
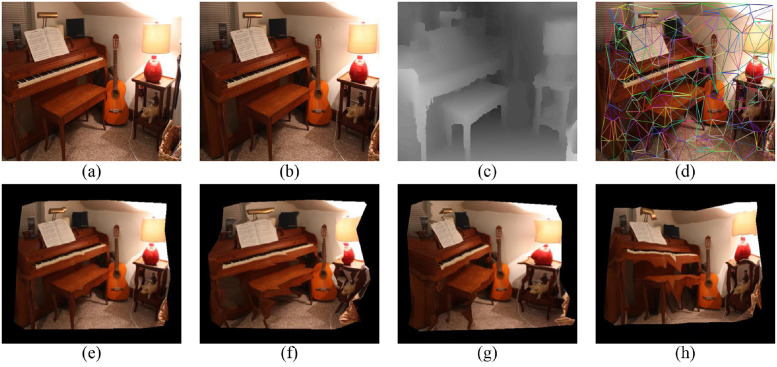
3D reconstruction experiment of scene Piano. (a) left image, (b) right image, (c) disparity image, (d) Delaunay triangulation, (e) view 1, (f) view 2, (g) view 3, (h) view 4. (a) and (b) are republished from [vision.middlebury.edu/stereo/] under a CC BY license, with permission from [D. Scharstein], original copyright [2002].

**Fig 11:**
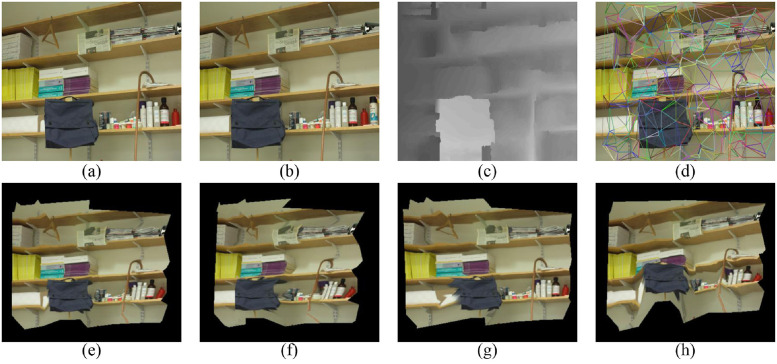
3D reconstruction experiment of scene Shelves. (a) left image, (b) right image, (c) disparity image, (d) Delaunay triangulation, (e) view 1, (f) view 2, (g) view 3, (h) view 4. (a) and (b) are republished from [vision.middlebury.edu/stereo/] under a CC BY license, with permission from [D. Scharstein], original copyright [2002].

In this section, six image pairs in the Middlebury dataset are selected to perform a 3D reconstruction experiment. The experimental results are shown in Figs 7–11, for the Cloth1, Wood1, Djembe, Piano, and Shelves scenes, respectively. In these figures, (a)-(d) are the left image, right image, disparity image, and Delaunay triangulation, while (e)-(h) are the 3D reconstruction results of these scenes from different angles. The 3D reconstruction results of scenes Cloth1 and Cloth3 are accurate, and the proposed method can accurately restore the surface of the cloth. The 3D reconstruction results of other scenes are not ideal due to the occurrence of depth discontinuity in these scenes.

**Fig 12:**
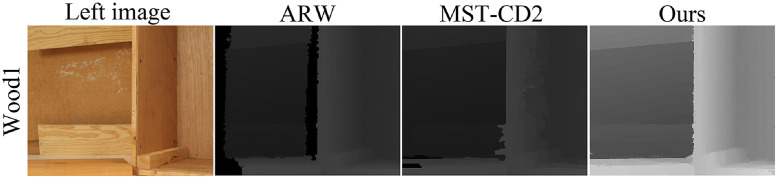
Wood1 scene in the Middlebury2 dataset. The left image in the figure is republished from [vision.middlebury.edu/stereo/] under a CC BY license, with permission from [D. Scharstein], original copyright [2002].

**Fig 13:**
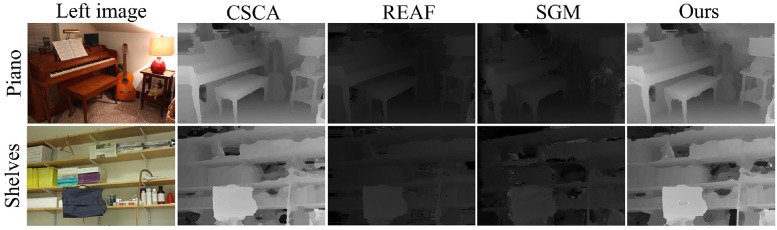
Piano scene and the Shelves scene in the Middlebury3 dataset. The left image in the figure is republished from [vision.middlebury.edu/stereo/] under a CC BY license, with permission from [D. Scharstein], original copyright [2002].

The proposed method was also compared with other semiglobal stereo matching methods and the comparison results are shown in Figs 12 and 13. We compare the disparity maps generated by different stereo matching methods as a qualitative analysis of 3D reconstruction because comparing the texture mapping models of different methods will make distinguishing the contrast method from the proposed method difficult. Fig 12 is the Wood1 scene in the Middlebury2 dataset, where the comparison method is ARW [32] and MST-CD2 [33]. Fig 13 shows the Piano scene and Shelves scene in of the Middlebury3 dataset, where the comparison method is CSCA [34], REAF [35] and SGM [12]. In these four scenes, the proposed method has more accurate disparity edges than other methods, so that regions of different depths can be distinguished more accurately.

The quantitative experimental results on Middlebury2 and Middlebury3 also show the superior performance of the proposed method. Three scenes (Cloth1, Cloth3 and Wood1) in the Middlebury2 dataset and three scenes (Djembe, Piano and Shelves) in the Middlebury3 dataset are used to conduct comparative experiments. We evaluate 3D reconstruction effects of the proposed method by calculating the average error distance between the point cloud generated by different methods and its ground truth. The ground truth of the point cloud is generated from the ground truth of the disparity map. Each scene in Middlebury2 and Middlebury3 contains more than 150,000 points and more than 350,000 points, respectively. Table 1 shows the experimental results. The data in Table 1 represent the average distance between each point and the ground truth in different scenes, in units of millimeters.

**Table 1:** Comparison of 3D reconstruction errors for six scenes in Middlebury2 and Middlebury3 (measurement unit: mm).

	Cloth1	Cloth3	Wood1	Djembe	Piano	Shelves
ARW [32]	**0.40**	**0.62**	1.11	0.83	2.54	1.66
CSCA [34]	1.69	1.12	0.71	1.12	2.46	6.59
MST-CD2 [33]	0.58	2.30	11.3	1.37	2.64	13.9
REAF [35]	31.0	1.95	22.5	6.26	11.1	21.2
SGM [12]	16.8	13.7	17.9	8.53	11.9	20.5
Proposed Approach	0.68	1.31	**0.59**	**0.00**	**1.82**	**1.47**

### 4.2 KITTI dataset

**Fig 14:**
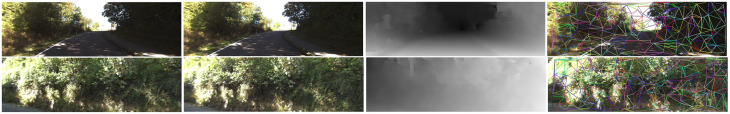
3D reconstruction experiment of scenes in KITTI 2012. It can be downloaded via https://osf.io/7srj4.

**Fig 15:**
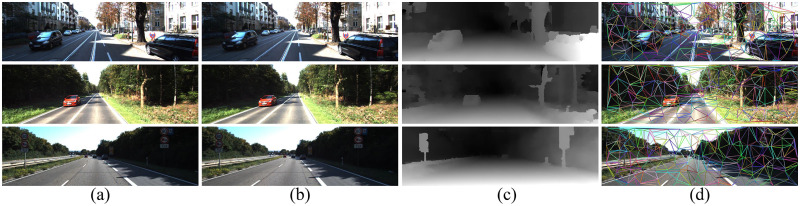
3D reconstruction experiment of scenes in KITTI 2015. It can be downloaded via https://osf.io/9kzma.

The proposed method was evaluated on KITTI 2012 and KITTI 2015 benchmarks, and compared with other methods. Since the scene of the KITTI dataset is an outdoor environment, texture mapping was not implemented. Implementing texture mapping in places where the depth is not continuous will distort the reconstruction results. Fig 14 shows the three scenes in KITTI 2012, which can be downloaded via https://osf.io/7srj4. Table 2 is the evaluation result of the proposed method on the KITTI 2012 benchmark. Compared with other semiglobal stereo matching methods, the proposed second-order semiglobal stereo matching method has advantages in various evaluation indicators. Fig 15 shows the three scenes in KITTI 2015, which can be downloaded via https://osf.io/9kzma. Table 3 shows the evaluation results of the proposed method on the KITTI 2015 benchmark.

**Table 2:** Depth information of 3D reconstruction in the KITTI 2012 benchmark.

	Out-Noc(%)	Out-All(%)	Avg-Noc(px)	Avg-All(px)
ARW [32]	5.2	6.87	1.2	1.5
SGM [12]	5.76	7.0	1.2	1.3
iSGM [36]	5.11	7.15	1.2	2.1
wSGM [37]	4.97	6.18	1.3	1.6
pSGM [38]	4.68	6.13	1.0	1.4
Proposed Approach	**3.45**	**4.22**	**0.9**	**1.0**

**Table 3:** Depth information of 3D reconstruction in the KITTI 2015 benchmark.

	D1-bg(%)	D1-fg(%)	D1-all(%)
REAF [35]	8.43	18.51	10.11
SGM [12]	5.15	15.29	6.84
pSGM [38]	4.84	11.64	5.97
SPS+FF++ [39]	5.47	12.19	6.59
Proposed Approach	**4.08**	**11.07**	**5.24**

### 4.3 Results of Delaunay triangulation based on fast point positioning

The proposed Delaunay triangulation based on fast point positioning was evaluated on the Middlebury dataset and the KITTI dataset. Table 4 shows the experimental results. The original Delaunay triangulation based on point-by-point insertion uses the implementation in OpenCV, which is denoted as Ori-D in Table 4.

**Table 4:** Results of Delaunay triangulation based on fast point positioning (measurement unit: ms).

	K-12(*F*_*avg*_:115)	K-15(*F*_*avg*_:115)	M-2(*F*_*avg*_:95)	M-3(*F*_*avg*_:124)
Ori-D	2.59	2.45	1.87	3.26
Fast-D	**0.08**	**0.12**	**0.13**	**0.17**

In Table 4, *F*_*avg*_ represents the average number of feature points of each image, which is the average size of the point set processed by Delaunay triangulation. K-12 represents the KITTI 2012 dataset, which contains 388 images; K-15 represents the KITTI 2015 dataset, which contains 398 images; M-2 represents the Middlebury2 dataset, which contains 31 images; M-3 represents the Middlebury3 dataset, which contains 23 images. Fast-D in Table 4 represents the proposed approach. Compared with the original Delaunay triangulation based on point-by-point insertion, the proposed fast point positioning method is significantly improved in terms of time efficiency. When processing a large amount of data (for example, 1000 points), the proposed method processes the data within 1 ms, but the Delaunay triangulation implementation in OpenCV cannot handle the data amount.

## 5 Conclusions

3D reconstruction based on binocular vision combines camera parameters and disparity maps to calculate all of the point coordinates in a scene and perform a surface reconstruction of the point cloud. In this paper, a 3D reconstruction method based on binocular stereo matching and fast point positioning of Delaunay triangulation is proposed to improve the accuracy and efficiency of 3D reconstruction. First, a second-order semiglobal stereo matching method combining multiple matching costs is used to obtain a more robust disparity map. Second, the 3D point cloud is obtained with camera parameters and disparity maps, which is smoothed by guided filtering to remove the noise points and retain details. Finally, the Delaunay triangulation method based on fast point positioning is used to reconstruct the surface of the point cloud and improve the visibility of the 3D model.

The Middlebury and KITTI datasets are used to evaluate the performance of the proposed method. The proposed method can obtain accurate depth information and reconstruct the surface of the point cloud to obtain a 3D model. The depth information obtained thus far only comes from visible light images. In future work, we will combine the accurate 3D information of LIDAR and the guidance of visible light images to achieve high-precision 3D information perception. To further improve the authenticity of the 3D model, we will also study triangulation in cases with shadows and occlusions in the future.

## Supporting information

S1 Dataset (EML)

S2 Dataset (EML)

S3 Dataset (EML)

S1 File (PDF)

S2 File (ZIP)
