# Supplementary figures and images for: 3D reconstruction method based on second-order semiglobal stereo matching and fast point positioning Delaunay triangulation
Source: PLoS One. 2022 Jan 25;17(1):e0260466. doi: 10.1371/journal.pone.0260466 (PMC8789135; doi:10.1371/journal.pone.0260466)

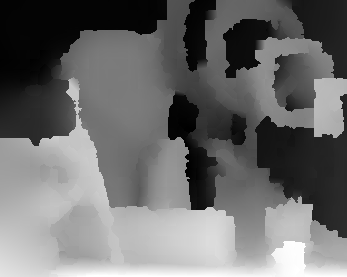

Supplement: S2 File — (ZIP) [file pone.0260466.s005.zip › Supplementary materials/3D Reconstruction Results/ArtL/disp.png]

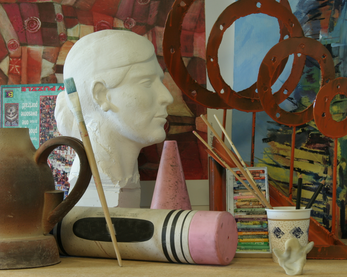

Supplement: S2 File — (ZIP) [file pone.0260466.s005.zip › Supplementary materials/3D Reconstruction Results/ArtL/im0.png]

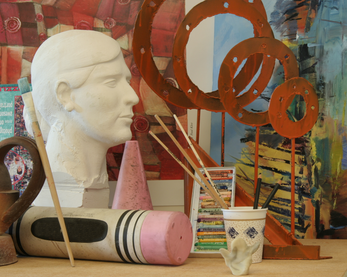

Supplement: S2 File — (ZIP) [file pone.0260466.s005.zip › Supplementary materials/3D Reconstruction Results/ArtL/im1.png]

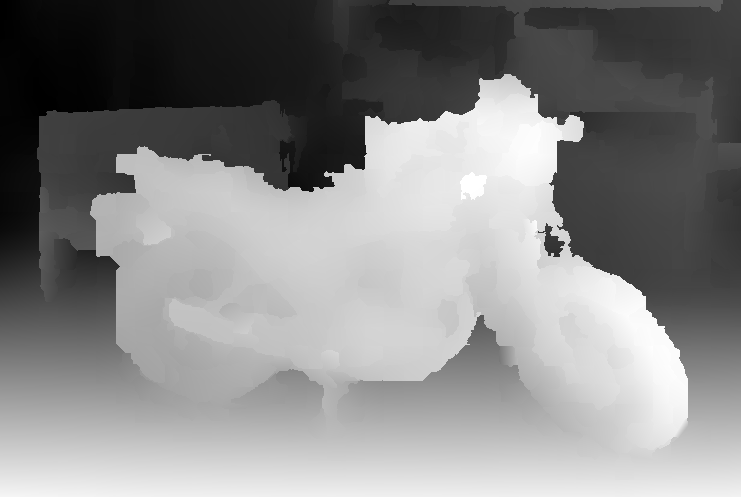

Supplement: S2 File — (ZIP) [file pone.0260466.s005.zip › Supplementary materials/3D Reconstruction Results/Motorcycle/disp.png]

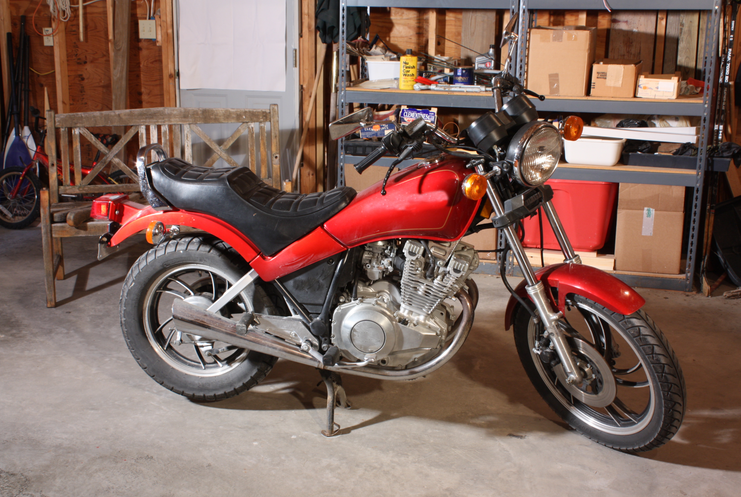

Supplement: S2 File — (ZIP) [file pone.0260466.s005.zip › Supplementary materials/3D Reconstruction Results/Motorcycle/im0.png]

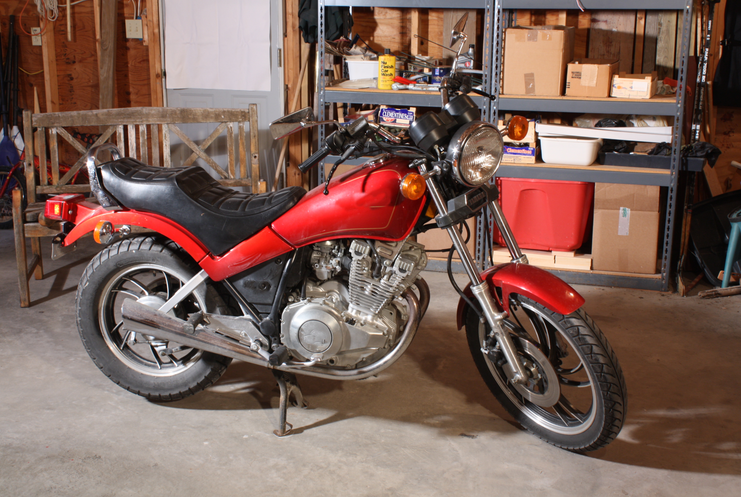

Supplement: S2 File — (ZIP) [file pone.0260466.s005.zip › Supplementary materials/3D Reconstruction Results/Motorcycle/im1.png]

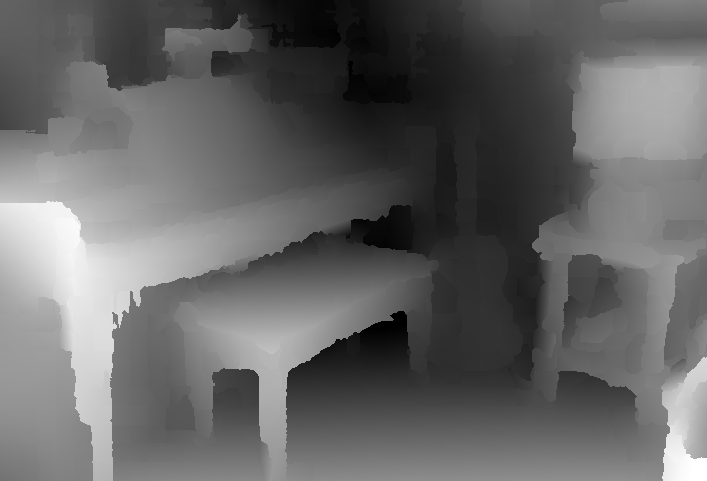

Supplement: S2 File — (ZIP) [file pone.0260466.s005.zip › Supplementary materials/3D Reconstruction Results/Piano/disp.png]

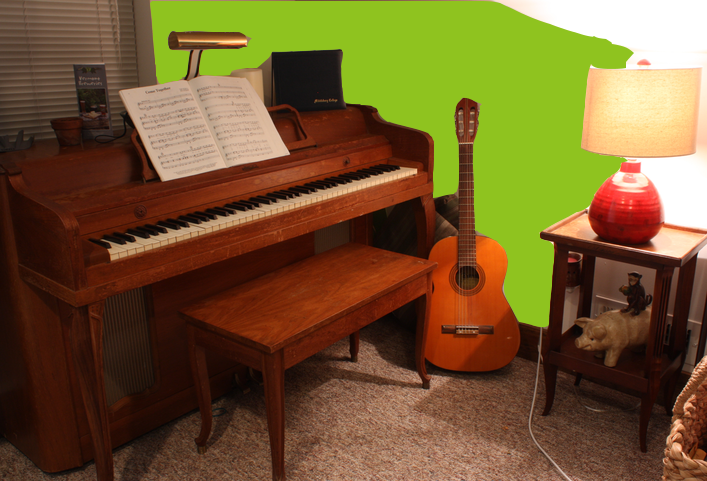

Supplement: S2 File — (ZIP) [file pone.0260466.s005.zip › Supplementary materials/3D Reconstruction Results/Piano/im0.png]

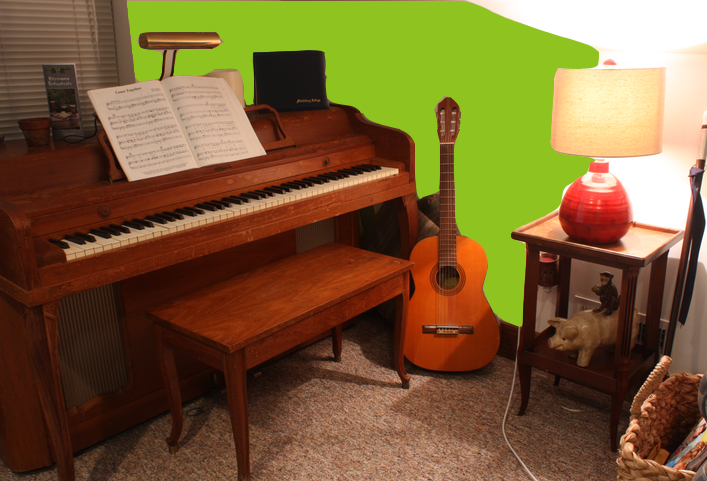

Supplement: S2 File — (ZIP) [file pone.0260466.s005.zip › Supplementary materials/3D Reconstruction Results/Piano/im1.png]

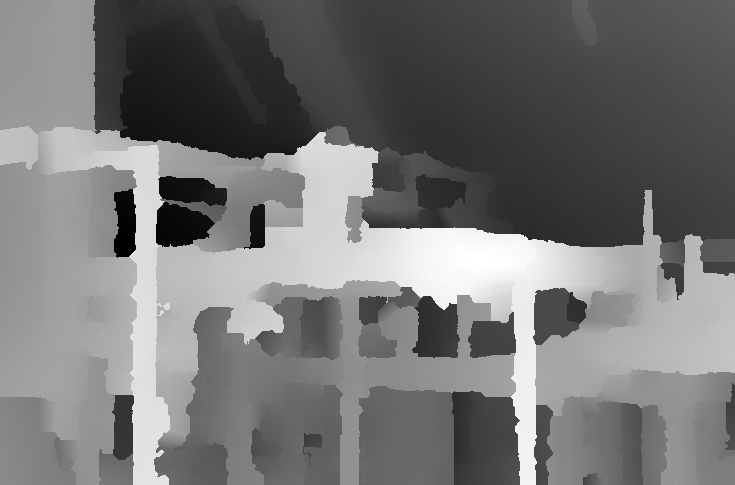

Supplement: S2 File — (ZIP) [file pone.0260466.s005.zip › Supplementary materials/3D Reconstruction Results/Pipes/disp.png]

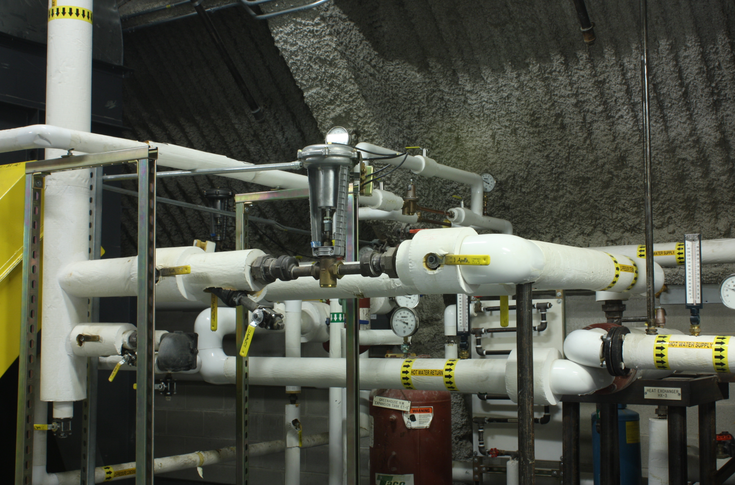

Supplement: S2 File — (ZIP) [file pone.0260466.s005.zip › Supplementary materials/3D Reconstruction Results/Pipes/im0.png]

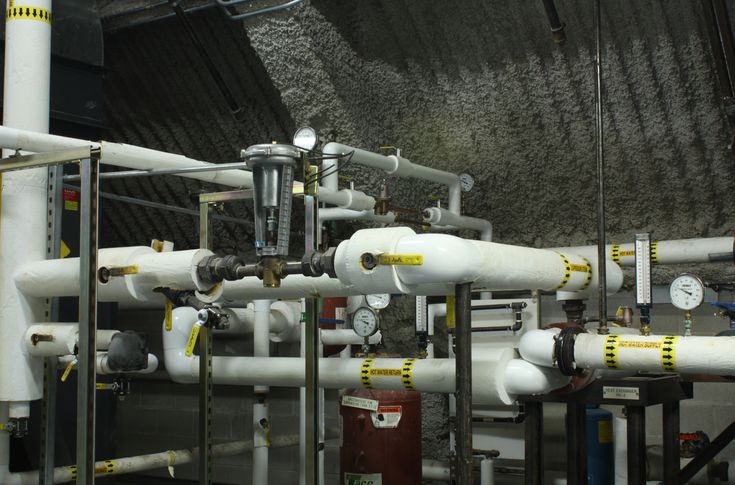

Supplement: S2 File — (ZIP) [file pone.0260466.s005.zip › Supplementary materials/3D Reconstruction Results/Pipes/im1.png]

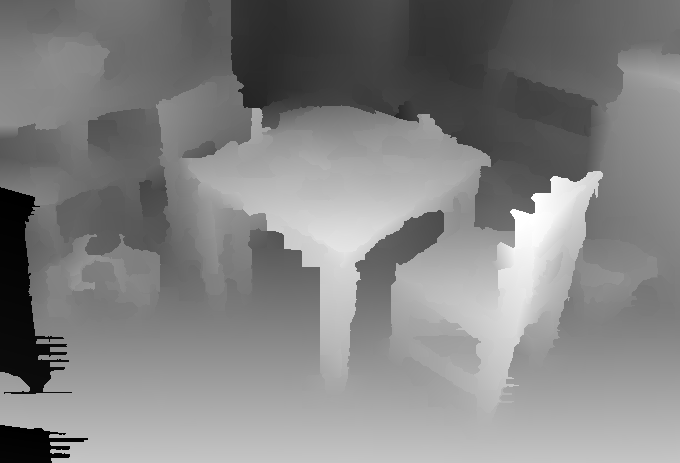

Supplement: S2 File — (ZIP) [file pone.0260466.s005.zip › Supplementary materials/3D Reconstruction Results/Playtable/disp.png]

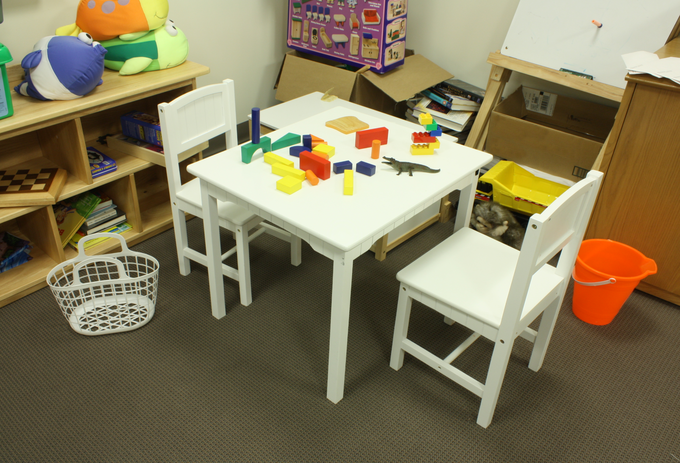

Supplement: S2 File — (ZIP) [file pone.0260466.s005.zip › Supplementary materials/3D Reconstruction Results/Playtable/im0.png]

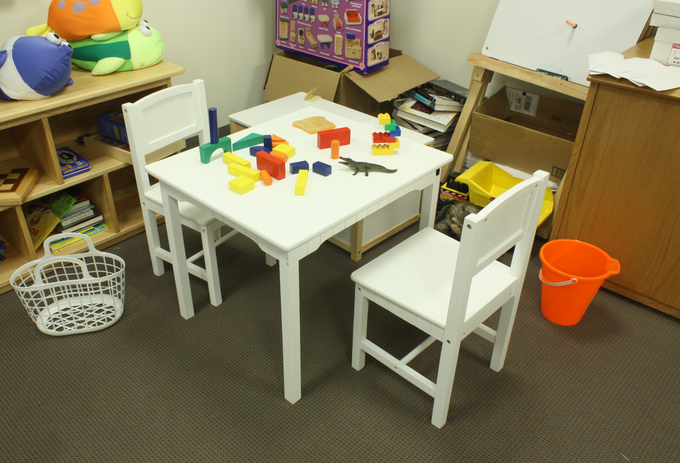

Supplement: S2 File — (ZIP) [file pone.0260466.s005.zip › Supplementary materials/3D Reconstruction Results/Playtable/im1.png]

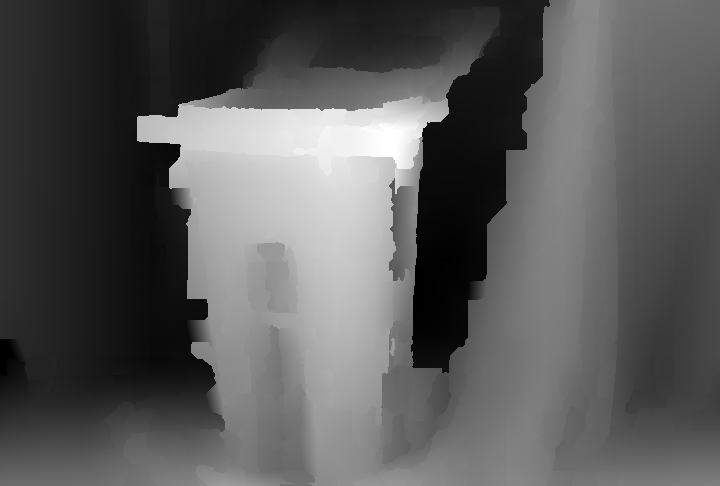

Supplement: S2 File — (ZIP) [file pone.0260466.s005.zip › Supplementary materials/3D Reconstruction Results/Recycle/disp.png]

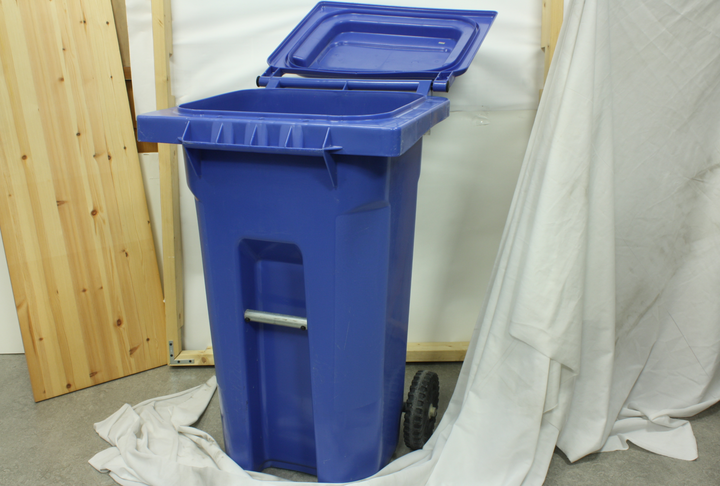

Supplement: S2 File — (ZIP) [file pone.0260466.s005.zip › Supplementary materials/3D Reconstruction Results/Recycle/im0.png]

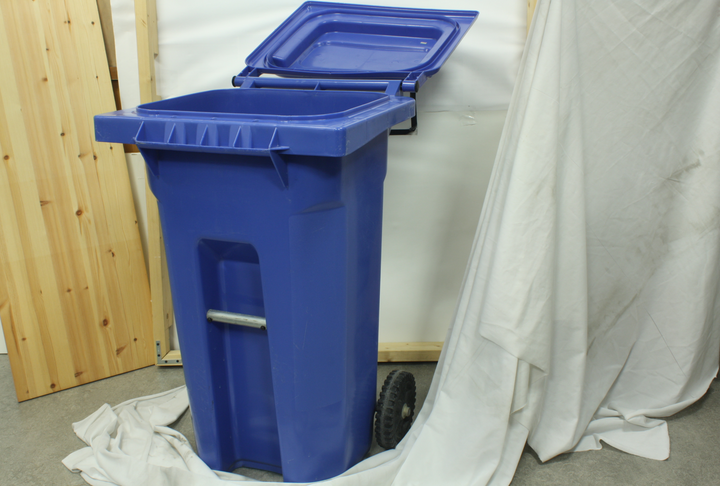

Supplement: S2 File — (ZIP) [file pone.0260466.s005.zip › Supplementary materials/3D Reconstruction Results/Recycle/im1.png]

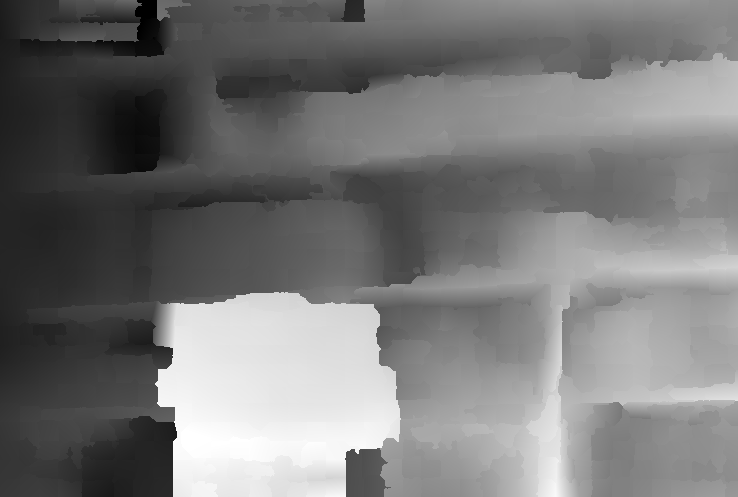

Supplement: S2 File — (ZIP) [file pone.0260466.s005.zip › Supplementary materials/3D Reconstruction Results/Shelves/disp.png]

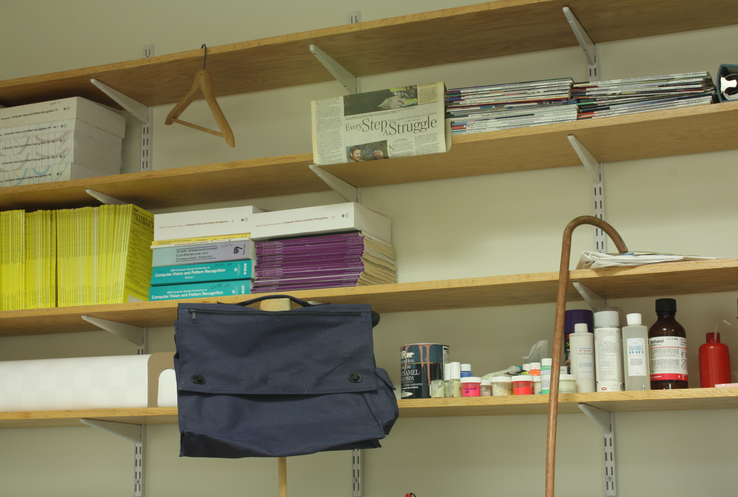

Supplement: S2 File — (ZIP) [file pone.0260466.s005.zip › Supplementary materials/3D Reconstruction Results/Shelves/im0.png]

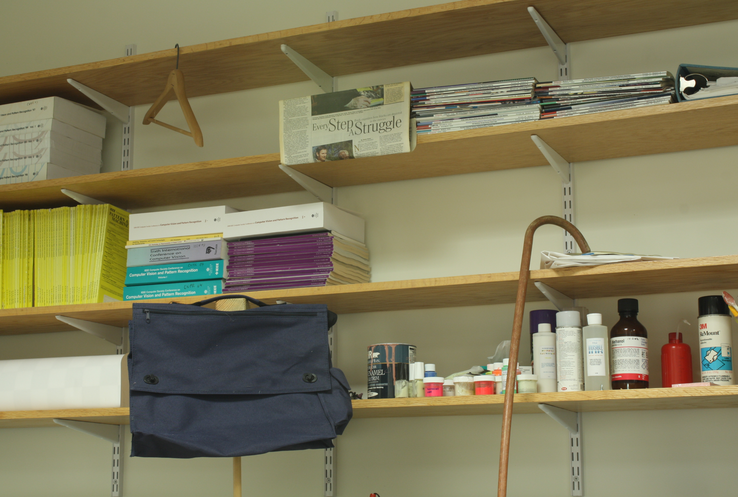

Supplement: S2 File — (ZIP) [file pone.0260466.s005.zip › Supplementary materials/3D Reconstruction Results/Shelves/im1.png]

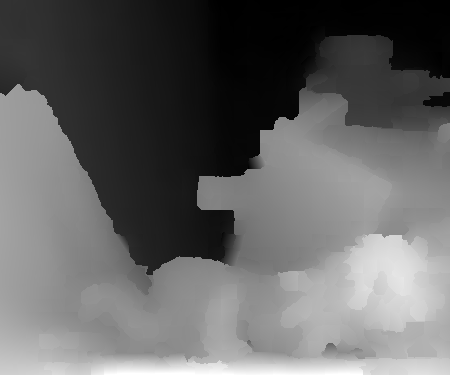

Supplement: S2 File — (ZIP) [file pone.0260466.s005.zip › Supplementary materials/3D Reconstruction Results/Teddy/disp.png]

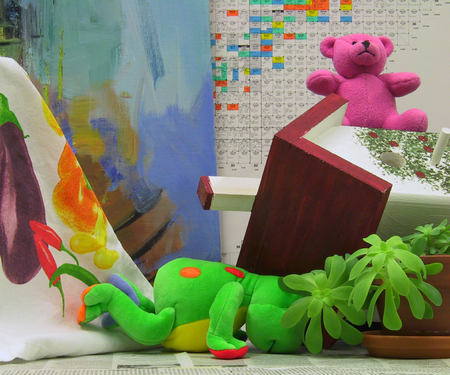

Supplement: S2 File — (ZIP) [file pone.0260466.s005.zip › Supplementary materials/3D Reconstruction Results/Teddy/im0.png]

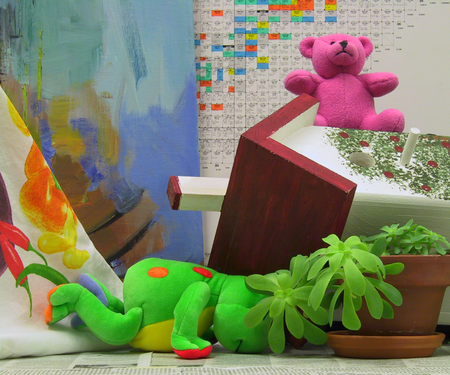

Supplement: S2 File — (ZIP) [file pone.0260466.s005.zip › Supplementary materials/3D Reconstruction Results/Teddy/im1.png]

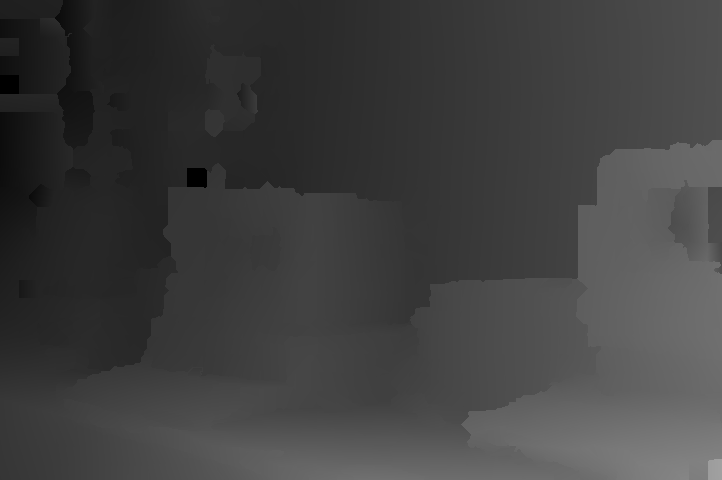

Supplement: S2 File — (ZIP) [file pone.0260466.s005.zip › Supplementary materials/3D Reconstruction Results/Vintage/disp.png]

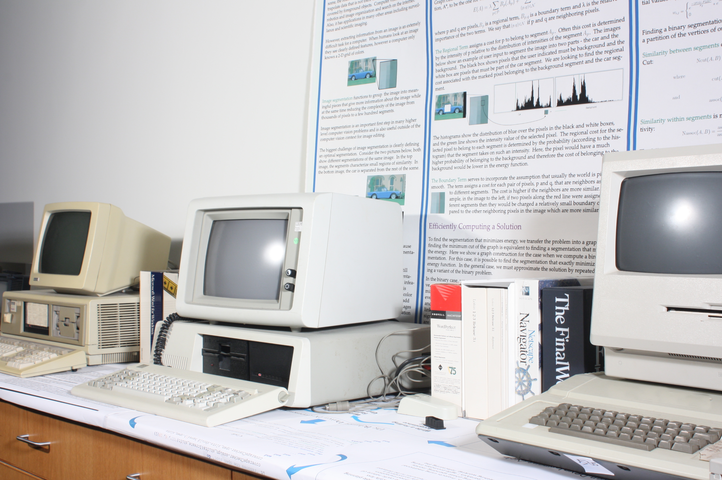

Supplement: S2 File — (ZIP) [file pone.0260466.s005.zip › Supplementary materials/3D Reconstruction Results/Vintage/im0.png]

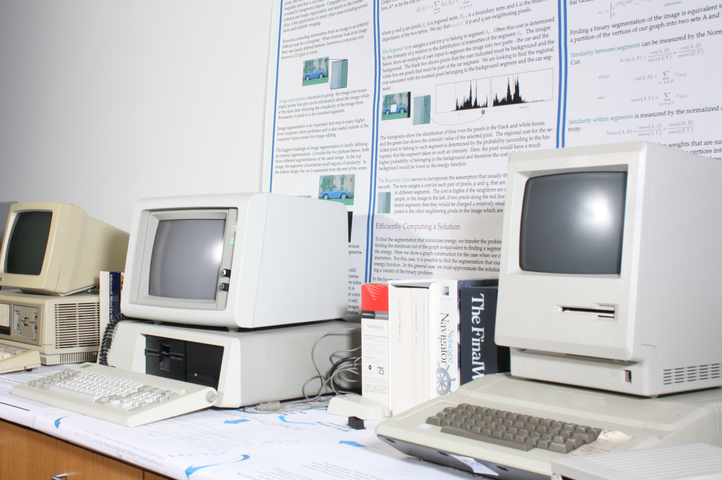

Supplement: S2 File — (ZIP) [file pone.0260466.s005.zip › Supplementary materials/3D Reconstruction Results/Vintage/im1.png]
